# The palm oil industry and noncommunicable diseases

**DOI:** 10.2471/BLT.18.220434

**Published:** 2018-01-08

**Authors:** Sowmya Kadandale, Robert Marten, Richard Smith

**Affiliations:** a United Nations Children’s Fund, World Trade Centre Block 6 (10th Floor), Jalan Jenderal Sudirman Kav. 29-31, Jakarta 12920, Indonesia.; b Department of Global Health and Development, The London School of Hygiene & Tropical Medicine, London, England.; c College of Medicine and Health, University of Exeter, Exeter, England.

## Abstract

Large-scale industries do not operate in isolation, but have tangible impacts on human and planetary health. An often overlooked actor in the fight against noncommunicable diseases is the palm oil industry. The dominance of palm oil in the food processing industry makes it the world’s most widely produced vegetable oil. We applied the commercial determinants of health framework to analyse the palm oil industry. We highlight the industry’s mutually profitable relationship with the processed food industry and its impact on human and planetary health, including detrimental cultivation practices that are linked to respiratory illnesses, deforestation, loss of biodiversity and pollution. This analysis illustrates many parallels to the contested nature of practices adopted by the alcohol and tobacco industries. The article concludes with suggested actions for researchers, policy-makers and the global health community to address and mitigate the negative impacts of the palm oil industry on human and planetary health.

## Résumé

Les grandes industries ne fonctionnent pas en vase clos; elles ont des effets tangibles sur la santé des individus et de la planète. Un acteur souvent négligé dans la lutte contre les maladies non transmissibles est l'industrie de l'huile de palme. Étant donné sa prédominance dans l'industrie alimentaire, l'huile de palme est l'huile végétale la plus largement produite au monde. Nous avons appliqué les déterminants commerciaux de la santé pour analyser l'industrie de l'huile de palme. Nous mettons en évidence la relation mutuellement profitable entre cette industrie et l'industrie des aliments transformés ainsi que ses effets sur la santé des individus et de la planète, et notamment des pratiques culturales néfastes liées à des maladies respiratoires, la déforestation, la diminution de la diversité biologique et la pollution. Cette analyse établit de nombreux parallèles avec la nature contestée de pratiques adoptées par les industries de l'alcool et du tabac. L'article se conclut par des propositions d’action pour que les chercheurs, les responsables politiques et la communauté mondiale de la santé examinent et atténuent les effets négatifs de l'industrie de l'huile de palme sur la santé des individus et de la planète.

## Resumen

Las industrias a gran escala no operan de forma aislada, sino que tienen un impacto tangible en la salud humana y del planeta. Un agente que a menudo se ignora en la lucha contra las enfermedades no contagiosas es la industria del aceite de palma. El predominio del aceite de palma en la industria del procesamiento de alimentos lo convierte en el aceite vegetal más producido del mundo. Se han aplicado los determinantes comerciales del marco de la salud para analizar la industria del aceite de palma. Cabe destacar la relación mutuamente beneficiosa de la industria con la industria de los alimentos procesados y su impacto en la salud humana y del planeta, incluidas las prácticas de cultivo perjudiciales que están relacionadas con las enfermedades respiratorias, la deforestación, la pérdida de biodiversidad y la contaminación. Este análisis ilustra muchos paralelismos con la naturaleza controvertida de las prácticas adoptadas por las industrias del alcohol y el tabaco. El artículo concluye con varias sugerencias de posibles medidas para que los investigadores, los responsables de la formulación de políticas y la comunidad mundial de la salud aborden y mitiguen los impactos negativos de la industria del aceite de palma en la salud humana y del planeta.

## ملخص

لا تعمل الصناعات واسعة النطاق في عزلة، بل يكون لها تأثيرات ملموسة على صحة الإنسان وسلامة الكوكب. من بين العوامل التي غالبا ما يتم إغفالها في مكافحة الأمراض غير السارية، هي صناعة زيت النخيل. إن سيطرة زيت النخيل في صناعة تجهيز الأغذية تجعله الزيت النباتي الأكثر إنتاجًا في العالم. لقد قمنا بتطبيق العناصر التجارية المحددة لإطار العمل الصحي لتحليل صناعة زيت النخيل. كما نقوم بتسليط الضوء على علاقة مربحة للطرفين في هذه الصناعة مع صناعة الأغذية المصنعة، وأثرها على صحة الإنسان وسلامة الكوكب، بما يشمل الممارسات الزراعية الضارة التي ترتبط بأمراض الجهاز التنفسي، وإزالة الغابات، وفقدان التنوع البيولوجي، والتلوث. يوضح هذا التحليل العديد من أوجه التشابه بين الطبيعة المتنازع عليها للممارسات التي تنتهجها صناعات الكحوليات والتبغ. ويختتم المقال بإجراءات مقترحة للباحثين وواضعي السياسات والمجتمع الصحي العالمي للتعامل مع الآثار السلبية لصناعة زيت النخيل على صحة الإنسان والكوكب، والتخفيف منها.

## 摘要

大规模行业通常不是孤立进行的，相反地，它会对人类与地球健康产生切实影响。在与非传染性疾病作斗争的过程中，棕榈油行业通常会被忽视。棕榈油在食品加工行业占据主导地位，是目前世界上生产量最大的植物油品种。我们采用影响健康的商业因素框架来分析棕榈油行业。我们强调该行业与食品加工行业之间的互惠互利关系及其对人类和地球健康所产生的影响，包括与呼吸系统疾病、森林砍伐、生物多样性丧失和污染有关的有害耕作实践。该分析表明酒精和烟草行业所采用的实践与其争议性的本质存在诸多相似之处。本文为研究人员、政策制定者和全球卫生社区工作者总结了建议行动方案，以解决和减轻棕榈油行业对人类和地球健康带来的负面影响。

## Резюме

Крупные предприятия функционируют не в изоляции, и результаты их деятельности оказывают ощутимое воздействие на здоровье и благополучие человека и планеты. Одним из факторов, который часто упускают из вида в борьбе с неинфекционными заболеваниями, является производство пальмового масла. Преобладание пальмового масла в пищевой промышленности делает его наиболее распространенным растительным маслом в мире. Для анализа производства пальмового масла авторы использовали коммерческие детерминанты системы здравоохранения. Авторы подчеркивают взаимовыгодную связь этой индустрии с пищевой промышленностью и ее влияние на здоровье и благополучие человека и планеты, включая методы культивирования, имеющие такие негативные последствия, как респираторные заболевания, уничтожение лесов, сокращение биологического разнообразия и загрязнение окружающей среды. Этот анализ иллюстрирует многие параллели со спорным характером практик, применяемых в алкогольной и табачной промышленности. В статье авторы предлагают исследователям, высшему руководству и мировому сообществу специалистов здравоохранения действия по устранению и смягчению негативных последствий производства пальмового масла для здоровья и благополучия человека и планеты.

## Introduction

Public health discourse increasingly focuses on the role of alcohol, tobacco and sugar in the growing burden of noncommunicable diseases. Increasingly this dialogue highlights how, in the pursuit of increased profits, the industries involved in these products aim to shape public and political opinion as well as influence research outcomes to influence policies that endanger public health.^1^^,^^2^ The palm oil industry is missing from this dialogue.

Palm oil is one of the world’s most commonly used vegetable oils, present in around half of frequently used food and consumer products, from snacks to cosmetics.^3^^,^^4^ Worldwide production of the oil has increased from 15 million tonnes in 1995 to 66 million tonnes in 2017. The rapid expansion in use is attributed to yields nearly four times other vegetable oil crops, with similar production costs; favourable characteristics for the food industry (its relatively high smoke point and being semisolid state at room temperature); and strategies aimed at ensuring government policies are supportive to the expansion of palm oil cultivation, production and use.^5^ While these factors associated with palm oil offer clear advantages for the processed food industry, the oil contains a much higher percentage of saturated fats compared to other vegetable oils.^6^ Although its negative health impacts are contested,^7^ a meta-analysis of increased palm oil consumption in 23 countries found a significant relationship with higher mortality from ischaemic heart disease.^8^ Another systematic review found that palm oil consumption increased blood levels of atherogenic low-density lipoprotein cholesterol.^6^ As early as 2003, the World Health Organization (WHO) and the Food and Agriculture Organization (FAO) described the evidence linking saturated fat consumption with increased risk of cardiovascular disease as convincing.^9^

The indirect health impacts of oil-palm cultivation are less contested; clearing land for plantations by slash-and-burn practices has led to recurring episodes of harmful haze in South-East Asia.^10^ The most recent occurrence, in 2015, led to an estimated 100 000 premature deaths in the region from pollutants and documented increases in respiratory, eye and skin diseases.^11^ The impact of the industry on planetary health, that is, “the health of human civilisation and the state of the natural systems on which it depends”,^12^ through the cultivation practices of oil-palm trees has also been well-documented. This entails large-scale deforestation, including loss of up to 50% of trees in some tropical forest areas; endangerment of at-risk species; increased greenhouse gas emissions (due to deforestation and drainage of peat bogs); water and soil pollution; and the rise of certain invasive species.^13^^,^^14^

Estimations suggest that more than two-thirds of the palm produced goes to food products, making the processed food industry’s relationship with the palm oil industry critical.^15^ With the United States Food and Drug Administration’s ban on trans-fatty acids (TFA) due to their potential adverse health impacts in 2015,^16^ and a similar recommendation by the WHO in 2018,^17^ an increase in the use of palm oil as a potential replacement for TFA in ultra-processed foods could be anticipated. This paper aims to describe the relationship between the palm oil and processed food industries and how these interconnect with public and planetary health. Box 1 lists the key terminology in the palm oil industry.

Box 1 **Key terminology in the palm oil industry**

Haze: smoke from biomass burnings, where resulting fine particulate matter reduces air quality to hazardous levels.

Palm oil: palm oil is harvested from the fruit of oil-palm trees *(*species: *Elaeis guineensis*). Common alternative labels for palm oil include: vegetable oil, vegetable fat, palm kernel, palm kernel oil, palm fruit oil, palmate, palmitate, palmolein, glyceryl, stearate, stearic acid, elaeis guineensis, palmitic acid, palm stearine, palmitoyl oxostearamide, palmitoyl tetrapeptide-3, sodium laureth sulfate, sodium lauryl sulfate, sodium kernelate, sodium palm kernelate, sodium lauryl lactylate/sulfate, hyrated palm glycerides, etyl palmitate, octyl palmitate, palmityl alcohol.^18^

Slash and burn: method of farming where forests are cut and any residue is burnt.

Smoke point: temperature at which oil produces a continuous, clearly visible smoke. Important indicator of the stability of oil, a higher smoke point allows more versatility in cooking.

Trans fatty acids: type of unsaturated fat associated with raising low-density lipoprotein cholesterol that is known to increase the risk for heart disease and stroke.

Ultra-processed foods: processed substances extracted or refined from whole foods, (such as fruits, crops or grains) e.g. oils, hydrogenated oils and fats, flours and starches, variants of sugar, and cheap parts or remnants of animal foods usually with little nutritional value compared to the original whole food.^17^

## Approach

**Fig. 1:**
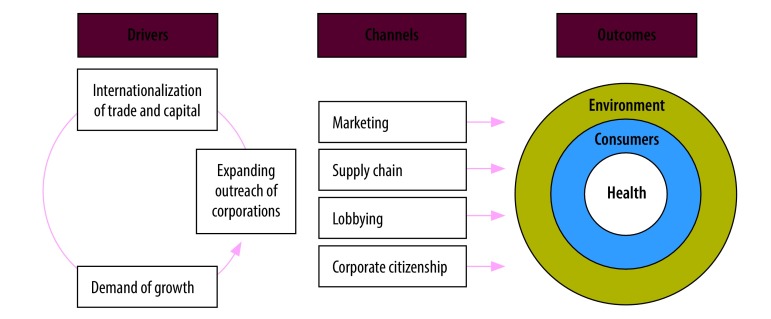
Commercial determinants of health

Source: Republished with permission from Kickbusch et al. 2016.^19^

The commercial determinants of health are defined as “strategies and approaches used by the private sector to promote products and choices that are detrimental to health.”^19^ We adapted a 2016 framework on the commercial determinants of health (Fig. 1) and applied it to the palm oil industry to review the three domains: (i) drivers (internationalization of trade and capital, expanding outreach of corporations and demands of economic growth); (ii) channels (marketing, supply chains, lobbying and corporate citizenship); and (iii) outcomes (on the environment, consumers and health). The environment component was adapted from the initial framework to expand the scope beyond the social environment.

## Drivers

### Internationalization of trade and capital

Oil-palm plantations cover over 27 million hectares worldwide, an area approximately the size of New Zealand. The industry is estimated to be worth 60 billion United States dollars (US$) and employs 6 million people,^7^ with an additional 11 million people indirectly dependent on it, particularly in rural areas where jobs can be scarce. In 2014, Indonesia and Malaysia accounted for over 53.3 million (85%) of the 62.4 million tonnes of global palm oil production and have rapidly expanded their farming and exports. Indonesia, for example, increased production from 19.2 tonnes in 2008 to 32.0 tonnes in 2016. The largest importers of palm oil are India, China, the European Union countries, Malaysia and Pakistan.^20^

The palm oil and processed food industries have mutually benefitted from increased sales and consumption of products through rapid internationalization and trade. This trend is likely to continue as low- and middle-income countries increasingly move from eating fresh, minimally processed foods to ultra-processed products.^21^ Sales by manufacturers of ultra-processed foods containing palm oil have been expanding.^22^

### Expanding outreach of corporations

Although many companies use palm oil, processing and refining is concentrated in a limited number of corporations. Companies source their supply from their own concessions, from a large number of third-party suppliers and smallholders, both independent and tied through partnership agreements.^23^ Increasingly, large corporations are expanding palm-oil refining capacity, expanding the scope of industry concentration.^24^ Indonesia and Malaysia have used government policies, including subsidies and land incentives, to assist industry expansion and facilitate greater investment.^23^

More than half the plantations in Indonesia are industrial estates of > 6000 hectares owned by private companies, with 40% smallholders with plantations < 25 hectares and 7% state-owned.^13^ When attempts are made to regulate oil-palm cultivation, industry leaders have highlighted the threat to smallholders’ livelihoods, making palm oil production a controversial political issue.^25^

### Demands of growth

The palm oil industry is projected to reach a production value of US$ 88 billion by 2022.^20^ The increasing availability of palm oil, alongside increasing numbers of countries banning TFA in processed foods,^26^^,^^27^ means that palm oil will likely remain the food industry’s preferred vegetable oil in ultra-processed foods. With China and India continuing to import palm oil for consumption, the growth in its use is anticipated to continue.

## Channels

### Marketing

Marketing of palm oil does not occur in the traditional sense. Responding to a backlash against accusations of poor environmental and labour practices, the industry has sought to portray its products as sustainable, while highlighting the contribution to poverty alleviation. For example, in advance of the European Union’s 2020 ban on palm oil as a biofuel, the industry launched advertisements featuring smallholder farmers whose livelihoods would be lost.^25^ There is also a mutual benefit for the palm oil and processed food industry, with the latter targeting advertisements for ultra-processed foods towards children (similar to efforts by the tobacco and alcohol industries in targeting children and adolescents)^28^^,^^29^ and the palm oil refining industry benefiting from the corresponding increase in sales of foods containing palm oil.^30^^–^^33^

### Supply chain

**Fig. 2:**
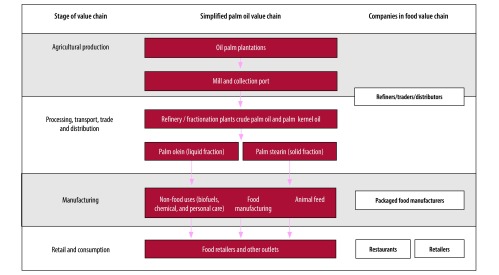
Global palm oil supply chain

Source: Adapted with permission from Ceres, 2018.^34^

The global palm oil supply chain has many businesses, systems and structures, making it difficult to draw a clear line between the different components and identify the impact of each actor.^23^ For example, a recent brief by the nongovernmental organization (NGO) Ceres, unpacks the key elements of the supply chain and the American industries and companies linked to them (Fig. 2).^34^ Unilever PLC, who claim to be the largest user of physically certified palm oil in the consumer goods industry,^35^ recently published details of its entire palm oil supply chain; this included 300 direct suppliers and 1400 mills used in its food, personal care and biofuel products.^26^^,^^27^ The scale of the supply chain is massive and, even by the company’s own admission, social and environmental issues persist.^26^ The supply chain demonstrates a strong association between the palm oil and processed food industries. Global food processing corporations are further venturing into palm oil refining, creating blurred lines across the supply chain, making it difficult to hold individual actors accountable for any adverse outcomes.

### Lobbying

Apart from establishing a strong lobbying presence in the European Union,^1^ the palm oil industry has fostered partnerships with policy and research institutes providing policy recommendations against regulation.^36^ For example, the industry-backed World Growth Institute criticised the World Bank's framework for palm-oil engagement – which seeks prioritisation of smallholders over large corporations and cultivation of plantations on degraded land instead of forested land – as 'anti-poor'.^37^ The palm oil industry has also sought to influence global health policy-making. For example, during the drafting of the 2003 WHO/FAO report on Diet, Nutrition and Prevention of Chronic Diseases, the Malaysian Palm Oil Promotion Council questioned the palm oil-related health concerns raised by the report and suggested that any efforts to curb consumption would threaten several million peoples’ livelihoods.^33^ These tactics, establishing lobbying structures in political and economic hubs, fighting regulations, attempting to undermine reliable sources of information and using poverty alleviation arguments, are similar to those pursued by the tobacco and alcohol industries.^38^^,^^39^

### Corporate citizenship

Several major companies and countries have joined to create industry associations to showcase their sustainability efforts. These are membership organizations composed of oil-palm growers, palm oil producers, consumer goods manufacturers, retailers, investors and NGOs which certify sustainability and fair labour standards and include entities such as the Roundtable on Sustainable Palm Oil and country-specific groups in Indonesia and Malaysia. In 2017, the Roundtable certified approximately 13.4 tonnes (approximately 20%) of the global production as sustainable. The Roundtable also has partnerships with the United Nations Economic and Social Council, United Nations Environment and United Nations Children’s Fund, aimed at improving its members’ business practices. Twelve of the 16 Roundtable board members are representatives of palm oil processers, manufacturers, retailers, banks, investors or international food processing companies. The sustainability certification effort has been linked to limited amounts of reduced deforestation, with a recent study finding little impact on forest loss and fire detection.^40^ Other studies have found that the Roundtable’s board members were still associated with companies involved in mass deforestation.^41^ Investigations by NGOs have found child labour and human rights violations at Roundtable members’ plantations.^42^

Despite some positive initiatives by the palm oil and processed food industries to cultivate, produce and source palm oil through sustainable, ethical practices, challenges remain. Agencies entering partnerships with industry-led initiatives are at risk of becoming complicit in detrimental practices. Indeed, NGOs such as Palm Oil Investigations withdrew support for the Roundtable after evidence of harmful business practices emerged.^43^

## Outcomes

**Fig. 3:**
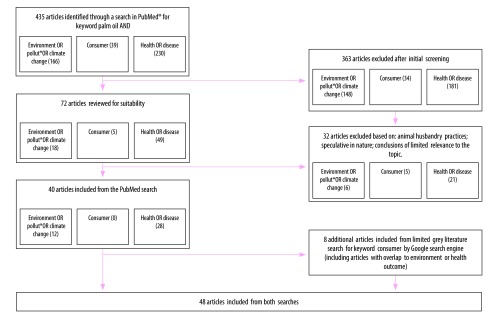
Flowchart of articles selected for the rapid review of the literature on the impact of palm oil on the environment, consumers and health

Given the importance of assessing the outcomes of the palm oil industry, we conducted a rapid review of the literature to better understand the impact on the environment, consumers and health. We made a keyword search initially via the PubMed® online database to identify peer-reviewed articles and subsequently via Google search engine to identify other sources of information (Box 2). The review was conducted in June and July 2018 and updated in October 2018. Of 435 articles identified and scanned, we included 40 peer-reviewed articles and eight articles from the grey literature (Fig. 3; Table 1).

Box 2 **Search strategy for the rapid review of the literature on the impact of palm oil on the environment, consumers and health**

We made an online search via the PubMed® database using the keyword “palm oil” in conjunction with relevant terms (AND “environment” OR ”pollution” OR ”climate change” OR “consumer” OR “health” OR “disease”). The review was conducted in June and July 2018 and updated in October 2018. The criteria for inclusion were articles published after 2000, in English language, of relevance to human health (through studies on humans or animal studies that drew conclusions on potential implications for human health), consumers or the environment. Articles were excluded if they were linked to animal husbandry practices, speculative in nature (e.g. profitability analyses), primarily aimed at industrial processes (e.g. monetizing palm oil mill effluent^a^) or drew conclusions of limited relevance to the topic (e.g. zoo-based conservation education).

While five articles initially appeared to be of relevance to palm oil and consumers, on further review, they were excluded. We therefore complemented the “consumer” keyword search with a review of the non-peer-reviewed literature, identified through search by the Google search engine using the same keywords. We limited the search to sources from governments, international agencies, NGOs and trusted media sources. Some of the results for “consumer” also yielded additional references relevant to environment and health, due to the intersection between human and planetary health, consumer practices and palm oil cultivation. Much of the grey literature related to consumers and the environment was focused on advocacy campaigns and calls for palm oil boycotts by NGOs and were therefore excluded as being beyond this paper’s scope.

NGO: nongovernmental organization.^a^ Highly polluting wastewater by-product of the palm oil production process.

**Table 1:** Articles included from a rapid review of the literature on palm oil and environment, consumer and health outcomes

Author, Year	Type of article	Title
Edem, 2002^44^	Review	Palm oil: biochemical, physiological, nutritional, hematological, and toxicological aspects: a review
Sundram et al., 2003^45^	Review^a^	Palm fruit chemistry and nutrition
World Health Organization-Food & Agriculture Organization, 2003^9^	Report	Joint WHO–FAO expert consultation on diet, nutrition, and the prevention of chronic diseases^b^
Fitzherbert et al., 2008^46^	Research	How will oil palm expansion affect biodiversity?
Mukherjee & Mitra, 2009^7^	Review	Health effects of palm oil
Oguntibeju et al., 2009 ^47^	Review	Red palm oil: nutritional, physiological and therapeutic roles in improving human wellbeing and quality of life
Bester et al., 2010^48^	Research	Cardiovascular effects of edible oils: a comparison between four popular edible oils
Hayes et al., 2010^49^	Review	Replacing trans fat: the argument for palm oil with a cautionary note on interesterification
Loganathan et al., 2010^50^	Review	Health promoting effects of phytonutrients found in palm oil
Oyewole et al., 2010^51^	Review	Public health nutrition concerns on consumption of red palm-oil (RPO): the scientific facts from literature
Persson et al., 2010^52^	Research	Preserving the world's tropical forests – a price on carbon may not do
Chen et al., 2011^8^	Systematic review	Multi-country analysis of palm oil consumption and cardiovascular disease mortality for countries at different stages of economic development: 1980–1997
Anonymous, 2012^53^	Commentary	The other oil problem. The world’s growing appetite for cheap palm oil is destroying rain forests and amplifying climate change
Downs et al., 2013^54^	Research	Reformulating partially hydrogenated vegetable oils to maximise health gains in India: is it feasible and will it meet consumer demand?
Fattore et al., 2014^55^	Systematic review	Palm oil and blood lipid–related markers of cardiovascular disease: a systematic review and meta-analysis of dietary intervention trials
Ho et al., 2014^56^	Research	Impact of 2013 South Asian haze crisis: study of physical and psychological symptoms and perceived dangerousness of pollution level
May et al., 2014^57^	Review^a^	Research advancements in palm oil nutrition: recent advancements in palm oil nutrition
Downs et al., 2015^58^	Research	The need for multisectoral food chain approaches to reduce trans fat consumption in India
Hosseini et al., 2015^14^	Research	Pollutant in palm oil production process
Mancini et al., 2015^59^	Review	Biological and nutritional properties of palm oil and palmitic acid: effects on health
Odia et al., 2015^60^	Review	Palm oil and the heart: a review
Sun et al., 2015^6^	Meta-analysis	Palm oil consumption increases LDL cholesterol compared with vegetable oils low in saturated fat in a meta-analysis of clinical trials
Whitmee et al., 2015^12^	Research	Safeguarding human health in the Anthropocene epoch: report of The Rockefeller Foundation–Lancet Commission on planetary health
Boateng et al., 2016^61^	Review	Coconut oil and palm oil's role in nutrition, health and national development: a review
Crippa et al., 2016^62^	Research	Population exposure to hazardous air quality due to the 2015 fires in Equatorial Asia
Koplitz et al., 2016^63^	Research	Public health impacts of the severe haze in Equatorial Asia in September–October 2015: demonstration of a new framework for informing fire management strategies to reduce downwind smoke exposure
UNICEF Indonesia, 2016^64^	Report	Palm oil and children in Indonesia: exploring the sector’s impact on children’s rights^b^
Vijay et al., 2016^5^	Research	The impacts of oil palm on recent deforestation and biodiversity loss
World Bank, 2016^11^	Report	The cost of fire: an economic analysis of Indonesia’s 2015 fire crisis^b^
Hermes, 2017^65^	Online media article	Lack of consumer demand for sustainable palm oil^b^
Karthik et al., 2017^10^	Research	Understanding the Southeast Asian haze
Lebbie et al., 2017^66^	Research	Predictors of hypertension in a population of undergraduate students in Sierra Leone
Loganathan et al., 2017^67^	Review^a^	Health-promoting effects of red palm oil: evidence from animal and human studies
Paddison, 2017^68^	Online media article	70% of brands in Malaysia and Singapore don’t disclose palm oil use^b^
Paterson et al., 2017^69^	Review	Climate change affecting oil palm agronomy, and oil palm cultivation increasing climate change, require amelioration
Shankar et al., 2017^70^	Stakeholder analysis	Policies for healthy and sustainable edible oil consumption: a stakeholder analysis for Thailand
Sheldon et al., 2017^71^	Research	The impact of Indonesian forest fires on Singaporean pollution and health
Singh et al., 2017^72^	Research	Prevalence and correlates of hypertension in a semi-rural population of Southern India
Coriakula et al., 2018^73^	Research	The development and implementation of a new import duty on palm oil to reduce non-communicable disease in Fiji
Di Genova et al., 2018^74^	Review	Pediatric age palm oil consumption
Gallardo et al., 2018^75^	Research	Current and future effects of global change on a hotspot's freshwater diversity
Ismail et al., 2018^76^	Systematic review	Systematic review of palm oil consumption and the risk of cardiovascular disease
Meijaard et al., 2018^13^	Situation analysis	Oil palm and biodiversity: a situation analysis by the IUCN Oil Palm Task Force
Nicholas et al., 2018^77^	Research	Palm oil in Myanmar: a spatiotemporal analysis of the effects of industrial farming on biodiversity loss
Sin Teh et al., 2018^78^	Review^a^	sn-2 hypothesis: a review of the effects of palm oil on blood lipid levels
Palm Oil Investigations, accessed 2018^79^	Online factsheet	Names for palm oil^b^
World Wildlife Fund, accessed 2018^80^	Online factsheet	Palm oil: The hidden truth lurking in your home^b^
World Wildlife Fund, accessed 2018^18^	Online factsheet	Which everyday products contain palm oil?^b^

^a^ Denotes articles authored by the Malaysian Palm Oil Board.

^b^ Denotes grey literature – all other articles are from peer-reviewed sources.

### Environment

Forest, peatland and biodiversity losses, increased greenhouse gas emissions and habitat fragmentation as well as pollution are environmental concerns continually linked to the palm oil industry.^5^^,^^10^^,^^12^^,^^46^^,^^52^^,^^53^^,^^63^^,^^69^^,^^75^^,^^77^ In response, countries including Indonesia and Malaysia are increasing industry regulation, seeking to prevent slash-and-burn practices and restoring peatlands.^11^ Although the results are limited, companies are attempting to engage in more sustainable palm oil cultivation and production practices.^13^ Nevertheless, plantations with palm sustainability certification only encompass a fifth of all oil-palm cultivation, certification does not yield the desired benefits and there is limited consumer demand for sustainable palm oil.^65^

### Consumers

In recent years, there have been campaigns by NGOs to increase consumer awareness about palm oil production practices, although success appears limited.^65^^,^^80^ From the processed food industry and health perspective, much work remains to be done. Palm oil derivatives in food, household and cosmetic products can be listed in any one or more of its 200 alternate names.^79^ Some countries such as Australia and New Zealand only require peanut, sesame and soy oils to be explicitly labelled, while palm oil can fall under a generic category of vegetable oil.^79^ The World Wildlife Fund lists more than 25 common alternatives to palm oil labelling found in food products (Box 1).^18^ With its inclusion in many everyday products, unclear food labelling and sometimes conflicting information on health impacts, it can be difficult to know how to identify palm oil in foods. Consumers may be unaware of what they are eating or its safety.

### Health

Reports of the health impacts of palm oil consumption in foods are mixed.^44^^,^^49^^,^^51^^,^^55^^,^^59^^,^^61^^,^^66^^,^^74^^,^^76^ Some studies link consumption of palm oil to increased ischaemic heart disease mortality, raised low-density lipoprotein cholesterol, increased risk of cardiovascular disease and other adverse effects.^6^^,^^8^^,^^9^ Other studies show no negative effects^7^ or even favourable health outcomes from palm oil consumption.^7^^,^^45^^,^^47^^,^^48^^,^^50^^,^^57^^,^^60^^,^^67^^,^^78^ Four of the nine studies in our literature search showing overwhelmingly positive health associations were authored by the Malaysian Palm Oil Board, again drawing parallels with the tobacco and alcohol industries^38^^,^^39^ and calling into question the credibility of claims in favour of increased palm oil consumption. The contested nature of the evidence suggests the need for independent, comprehensive studies of the health impact of palm oil consumption. Countries such as Fiji, India and Thailand have initiated policy dialogues and analyses aimed at better understanding the role of palm oil in diets and best approaches to reducing saturated fats in the food-chain, but these discussions are far from conclusive.^54^^,^^58^^,^^70^^,^^72^^,^^73^

More unequivocally, land-clearing practices for oil palm cultivation have major public health consequences. Since the 1990s, air pollution from slash-and-burn practices have affected the health of populations in South-East Asia, especially the most vulnerable groups of the population, such as infants and children.^11^^,^^56^ Haze episodes, even across country borders, have been linked to premature deaths and increased respiratory illness as well as cardiovascular diseases.^62^^,^^71^ Of major concern is the effect of exposure to particulate matter on fetal, infant and child mortality, as well as children’s cognitive, educational and economic attainment.^81^^,^^82^ The direct and indirect impact of the palm oil cultivation industry on children, including child labour practices, is especially concerning. In Indonesia, around half of 4 million people employed in the industry are estimated to be women. Even when they are not directly employed, children dependent on palm oil workers are adversely affected by inadequate maternity protection, low breastfeeding rates, lack of child-care opportunities, poor maternal health and nutrition, and difficultly in accessing education.^64^

## Discussion

This paper illustrates how the palm oil industry, in close connection with the processed food industry, impacts human and planetary health. The impact also cuts across other sectors, such as education, child protection, as well as having implications for gender-related policies and practices. A limitation of our rapid review is that not all the information from these industries is publicly available and, with limited peer-reviewed materials available on the palm oil industry, we included media reports, environmental activist web sites and other grey literature. This article is not meant to be exhaustive and therefore does not avert the need for an extensive systematic review of the human and planetary health outcomes of the palm oil industry, spanning other sectors such as labour, gender and use as biofuel.

The palm oil industry is an overlooked actor in discussions on noncommunicable diseases. The current widespread use of palm oil draws attention to the ultra-processed unhealthy food system and the need to deepen and expand existing research on the industry. However, we need to carefully consider practical policy options and their implications. For example, encouraging use of oils with lower saturated fat content in ultra-processed foods could have a greater detrimental impact on the environment than palm oil, through further deforestation and loss of biodiversity (given the need for more natural resources to cultivate such crops). Policy-makers may therefore need to consider ways to reduce the demand for oils more specifically and for unhealthy ultra-processed foods more broadly. Such actions would benefit not only the noncommunicable disease agenda, but also human and planetary health as part of the sustainable development goals (SDGs).

### Suggestions for action

Addressing the palm oil industry’s impact goes beyond a single industry, product or sector. Taking a multifaceted approach, we suggest three sets of actions for researchers, policy-makers and the global health community (NGOs and international organizations; Box 3).

Box 3 **Suggested actions to address the palm oil industry’s impact**

**Address impact on health**

Researchers

- Investigate the health impact of ultra-processed foods, including specific ingredients such as palm oil;
- study the long-term consequences of daily consumption of unhealthy, ultra-processed foods and their ingredients, including the effects on children; and
- research the effect of combinations of ingredients in ultra-processed foods.

Policy-makers

- Identify and address industries that adversely impact noncommunicable diseases and the broader human and planetary health agenda;
- develop and enforce stricter labelling requirements for ultra-processed foods, including listing of ingredients and their potential harmful effects;
- regulate the palm oil supply chains across sectors such as health, environment, labour, and child protection, including needed gender-related policies and practices; and
- consider measures to reduce the production and consumption of unhealthy, ultra-processed foods.

Global health community

- Tackle the issue of unhealthy mass-produced and processed foods and beverages synergistically instead of discretely by ingredient (e.g. palm oil, sugar, fats); and
- facilitate consumer awareness and action on the negative impacts of palm oil cultivation, production and consumption.

**Mitigate industry influences**

Researchers

- Drawing on experience with the tobacco and alcohol industries, understand and mitigate the influence of industries involved in palm oil production and manufactured foods; and
- exercise caution when engaging in research activities using funding from the palm oil and related industries.

Policy-makers

- Avoid the influence of lobbying by food industries whose practices adversely impact human and planetary health;
- develop and enforce strict regulations that avoid political patronage or related practices (i.e. elected officials sitting on industry boards); and
- introduce measures to reduce the population’s consumption of unhealthy, ultra-processed foods (e.g. by taxation, restricting advertising) and to increase the consumption of healthier, whole foods.

Global health community

- When considering partnerships with the palm oil industry or their related entities, ensure public health priorities are not co-opted by private sector agendas; and
- avoid the risk of perceived or real complicity, including avoiding funding or partnership opportunities for health that might come at the expense of other sectors such as environment or labour.

**Work across SDGs**

Researchers

- Study interlinkages across complex systems of the palm oil and related industries aimed at identifying cross-sectoral solutions.

Policy-makers

- Design policies that do not sacrifice longer-term health, environmental and social concerns for immediate economic gains and profits.

Global health community

- Identify allies across sectors such as environment, child protection, labour and gender that can join in evidence generation and advocacy around the detrimental impacts of palm oil on human and planetary health; and
- reform global health governance structures and funding mechanisms with the aim of promoting intersectoral action instead of narrow disease-specific programmes.

SDG: sustainable development goal.

#### Understand impact on health

We need to better understand and address the content, health impact and supply chains of palm oil products. The evidence on health remains mixed. Furthermore, the so-called cocktail effect remains unknown; individual ingredients of ultra-processed foods may be harmless alone, but consumed in combination, daily, could be damaging.^83^ This also includes understanding the associated supply chains and the needed accountability measures aimed at addressing potential determinantal actions from the palm oil and related industries.

#### Mitigate industry influences

We need to mitigate the influence of the palm oil and related industries on public health policies and programmes. The relationship between the palm oil and processed food industries, and the tactics they employ, resembles practices adopted by the tobacco and alcohol industries. However, the palm oil industry receives comparatively little scrutiny. Palm oil use will likely continue, given the relatively low production costs of palm oil, high profit margins of ultra-processed foods, abundant use of palm oil in processed foods and prevalence of palm oil use in several industries (without a current viable alternative). As seen with recent examples, the public health community, whether multilateral agencies^84^ or research institutes^85^, is not immune to industry influence. Political ties to industries merit further exploration.^86^

#### Work across the SDGs 

Palm oil use in ultra-processed foods follows a long, complex chain. Even as the direct health impact remains unclear, cultivation and production and related practices contribute to environmental pollution, respiratory illnesses and loss of biodiversity. Furthermore, with documented forced and child labour and human rights abuses, as well as gender-related issues, such as inadequate maternity protections in palm oil plantations, understanding and addressing the influence of the palm oil industry cuts across different sectors and different SDGs. Therefore, narrow, health-specific measures cannot be implemented in isolation.

## Conclusions

As the most prevalent vegetable oil in food manufacturing, palm oil is an integral component of the food supply chain. While the direct health effects of palm oil remain contested, the indirect health impacts of cultivating this product are many. Commercial determinants play a vital role in a complex system that leads to the production and consumption of foods detrimental to human health. The discourse on noncommunicable diseases and human health can no longer be separated from the dialogue on planetary health.
